# Crystal structure of 5-(1-benzo­furan-2-yl)-3-(4-methyl­phen­yl)-4,5-di­hydro-1,2-oxazol-5-ol

**DOI:** 10.1107/S2056989015011263

**Published:** 2015-06-17

**Authors:** A. J. Ravi, A. C. Vinayaka, S. Jeyaseelan, M. P. Sadashiva, H. C. Devarajegowda

**Affiliations:** a Department of Physics, Yuvaraja’s College (Constituent College), University of Mysore, Mysore 570 005, Karnataka, India; b Department of Studies in Chemistry, Manasagangotri, University of Mysore, Mysore 570 006, India; c Department of Physics, St. Philomena’s College, Mysore, India

**Keywords:** crystal structure, benzo­furan, 1,2-oxazole, alcohol, biological properties, pharmaceutical properties

## Abstract

In the title compound, C_18_H_15_NO_3_, the isoxazole moiety adopts a shallow envelope conformation, with the C atom bearing the OH group displaced by 0.148 (1) Å from the mean plane through the other four atoms. The mean plane of this ring (all atoms) subtends dihedral angles of 87.19 (6) and 15.51 (7)° with the benzo­furan ring system (r.m.s. deviation = 0.007 Å) and the 4-methylphenyl ring, respectively. In the crystal, mol­ecules are linked by O—H⋯N hydrogen bonds, generating [001] *C*(5) chains, with adjacent mol­ecules in the chain related by *c*-glide symmetry. Weak C—H⋯O inter­actions link the chains into a three-dimensional network.

## Related literature   

For the biological and pharmaceutical properties of isoxazoles, see: Eddington *et al.* (2002 ▸); Lee *et al.* (2009 ▸); Rozman *et al.* (2002 ▸); Shin *et al.* (2005 ▸).
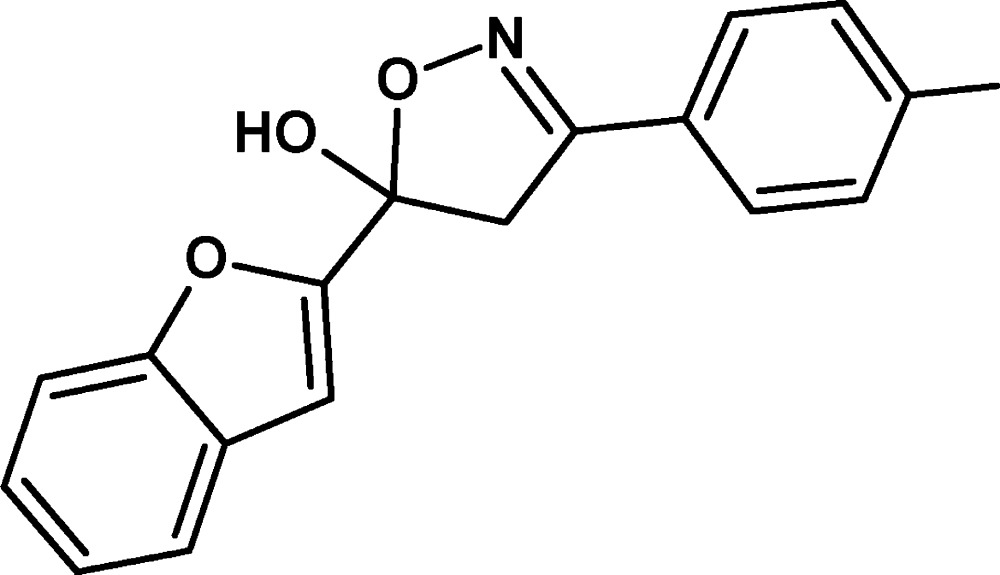


## Experimental   

### Crystal data  

C_18_H_15_NO_3_
*M* *_r_* = 293.31
Monoclinic, 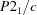

*a* = 10.2200 (15) Å
*b* = 14.2289 (19) Å
*c* = 10.2474 (15) Å
β = 93.058 (7)°
*V* = 1488.1 (4) Å^3^
*Z* = 4
Mo *K*α radiation
μ = 0.09 mm^−1^
*T* = 293 K
0.30 × 0.25 × 0.20 mm

### Data collection  

Bruker APEXII CCD diffractometer
23993 measured reflections
3452 independent reflections
2829 reflections with *I* > 2σ(*I*)
*R*_int_ = 0.047

### Refinement  

*R*[*F* ^2^ > 2σ(*F* ^2^)] = 0.044
*wR*(*F* ^2^) = 0.123
*S* = 1.03
3452 reflections
200 parameters
H-atom parameters constrained
Δρ_max_ = 0.24 e Å^−3^
Δρ_min_ = −0.18 e Å^−3^

### 

Data collection: *APEX2* (Bruker, 2009 ▸); cell refinement: *SAINT* (Bruker, 2009 ▸); data reduction: *SAINT*; program(s) used to solve structure: *SHELXS97* (Sheldrick, 2008 ▸); program(s) used to refine structure: *SHELXL97* (Sheldrick, 2008 ▸); molecular graphics: *PLATON* (Spek, 2009 ▸); software used to prepare material for publication: *SHELXL97*.

## Supplementary Material

Crystal structure: contains datablock(s) global, I. DOI: 10.1107/S2056989015011263/hb7438sup1.cif

Structure factors: contains datablock(s) I. DOI: 10.1107/S2056989015011263/hb7438Isup2.hkl

Supporting information file. DOI: 10.1107/S2056989015011263/hb7438Isup3.cml

**Fig. 1.**
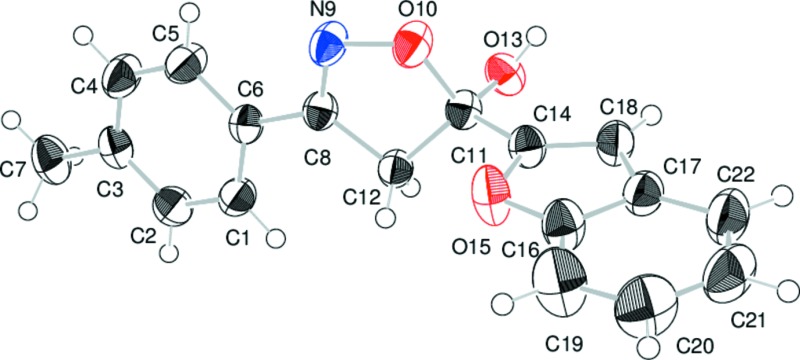
Perspective diagram of the molecule with 50% probability displacement ellipsoids.

**Fig. 2.**
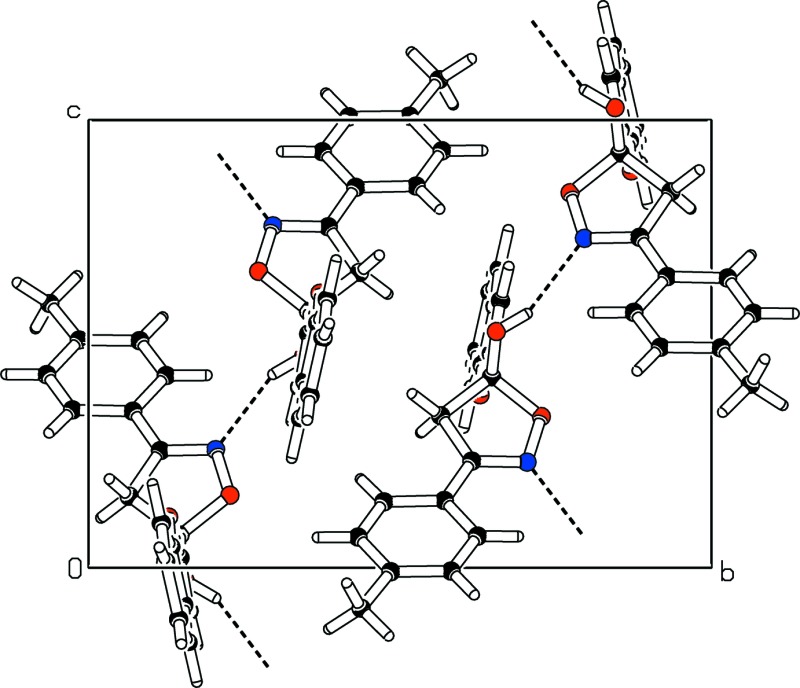
Packing diagram of the molecule viewed down the a axis.

. DOI: 10.1107/S2056989015011263/hb7438fig1.tif
Perspective diagram of the mol­ecule with 50% probability displacement ellipsoids.

a . DOI: 10.1107/S2056989015011263/hb7438fig2.tif
Packing diagram of the mol­ecule viewed down the *a* axis.

CCDC reference: 1405867

Additional supporting information:  crystallographic information; 3D view; checkCIF report

## Figures and Tables

**Table 1:** Hydrogen-bond geometry (, )

*D*H*A*	*D*H	H*A*	*D* *A*	*D*H*A*
O13H13N9^i^	0.82	2.17	2.9352(15)	156
C2H2O10^ii^	0.93	2.46	3.2328(17)	141
C7H7*C*O15^iii^	0.96	2.58	3.175(2)	121
C18H18O10^i^	0.93	2.54	3.4183(17)	158

Symmetry codes: (i) 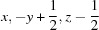; (ii) 
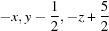
; (iii) 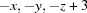.

## supplementary crystallographic information

### S1. Comment

Isoxazole derivatives bearing various substituents are known to have diverse biological and pharmaceutical activities; such as antiviral (Lee *et al.*, 2009), anti-HIV activities (Shin *et al.*, 2005) and anticonvulsant activity (Eddington *et al.*, 2002). In addition, isoxazole derivative is used for the treatment of rheumatoid arthritis (Rozman *et al.*, 2002). As part of our interest in these compounds, the title compound was chosen for the X-ray structure analysis.

In the molecular structure of the title compound (Fig. 1), the isoxazole moiety makes dihedral angles of 87.19 (6)° and 15.51 (7)° with benzofuran and phenyl ring, respectively. The dihedral angle between the benzofuran and phenyl ring is 81.67 (6)° The central isoxazole moiety adopts a slightly flattened envelope conformation with puckering parameter Q = 0.2406 (12) Å and φ = 183.41 (17)° , and the maximum deviation found on the puckered atom at C11 is -0.148 (1) Å. The crystal structure features C—H···O and O—H···N hydrogen bonds. The packing diagram of the molecule when viewed down the *a* axis as shown in Fig. 2.

### S2. Experimental

A solution of 2 Eq. of K_2_CO_3_ in water (1 ml) was added to the stirred solution of 4 Eq. of *N*-hydroxyl-4-toluenesulfonamide in MeOH/H_2_O (5/1 ml). Then 1 Eq. of 1-Benzofuran-2-yl-3-*p*-tolyl-propenone was added and reaction mixture was allowed to stir at room temperature for overnight. After completion of reaction, the reaction mixture was diluted with EtOAc, washed with water and brine. The organic extract was dried and concentrated under reduced pressure to give crude product, which was further purified using column chromatography (60–120 silica gel) to afford pure product.

### S3. Refinement

The H atoms were positioned geometrically and allowed to ride on their parent atom, with O–H distance is equal to 0.82 Å and C–H distance in the range of 0.93 to 0.97 Å; *U*_iso_(H) = 1.2–1.5*U*_eq_ (carrier atom) for all H atoms.

### Crystal data

**Table:**

C_18_H_15_NO_3_	*F*(000) = 616
*M**_r_* = 293.31	*D*_x_ = 1.309 Mg m^−^^3^
Monoclinic, *P*2_1_/*c*	Mo *K*α radiation, λ = 0.71073 Å
Hall symbol: -P 2ybc	Cell parameters from 3452 reflections
*a* = 10.2200 (15) Å	θ = 2.0–27.6°
*b* = 14.2289 (19) Å	µ = 0.09 mm^−^^1^
*c* = 10.2474 (15) Å	*T* = 293 K
β = 93.058 (7)°	Block, light yellow
*V* = 1488.1 (4) Å^3^	0.30 × 0.25 × 0.20 mm
*Z* = 4	

### Data collection

**Table:**

Bruker APEXII CCD diffractometer	*R*_int_ = 0.047
ω and φ scans	θ_max_ = 27.6°, θ_min_ = 2.0°
23993 measured reflections	*h* = −13→13
3452 independent reflections	*k* = −18→18
2829 reflections with *I* > 2σ(*I*)	*l* = −13→13

### Refinement

**Table:**

Refinement on *F*^2^	Primary atom site location: structure-invariant direct methods
Least-squares matrix: full	Secondary atom site location: difference Fourier map
*R*[*F*^2^ > 2σ(*F*^2^)] = 0.044	Hydrogen site location: inferred from neighbouring sites
*wR*(*F*^2^) = 0.123	H-atom parameters constrained
*S* = 1.03	*w* = 1/[σ^2^(*F*_o_^2^) + (0.0594*P*)^2^ + 0.3174*P*] where *P* = (*F*_o_^2^ + 2*F*_c_^2^)/3
3452 reflections	(Δ/σ)_max_ = 0.001
200 parameters	Δρ_max_ = 0.24 e Å^−^^3^
0 restraints	Δρ_min_ = −0.18 e Å^−^^3^

### Special details

**Table:**

Geometry. Bond distances, angles *etc*. have been calculated using the rounded fractional coordinates. All su's are estimated from the variances of the (full) variance-covariance matrix. The cell e.s.d.'s are taken into account in the estimation of distances, angles and torsion angles
Refinement. Refinement on *F*^2^ for ALL reflections except those flagged by the user for potential systematic errors. Weighted *R*-factors *wR* and all goodnesses of fit *S* are based on *F*^2^, conventional *R*-factors *R* are based on *F*, with *F* set to zero for negative *F*^2^. The observed criterion of *F*^2^ > σ(*F*^2^) is used only for calculating -*R*-factor-obs *etc*. and is not relevant to the choice of reflections for refinement. *R*-factors based on *F*^2^ are statistically about twice as large as those based on *F*, and *R*-factors based on ALL data will be even larger.

### Fractional atomic coordinates and isotropic or equivalent isotropic displacement parameters (Å^2^)

**Table:**

	*x*	*y*	*z*	*U*_iso_*/*U*_eq_	
O10	0.10420 (9)	0.22693 (6)	1.16214 (9)	0.0469 (3)	
O13	0.01366 (9)	0.15474 (6)	0.97218 (9)	0.0424 (3)	
O15	0.34411 (9)	0.12845 (9)	1.11345 (9)	0.0560 (4)	
N9	0.02005 (11)	0.20392 (8)	1.26398 (11)	0.0442 (3)	
C1	−0.10566 (14)	−0.02593 (9)	1.35272 (13)	0.0444 (4)	
C2	−0.19545 (15)	−0.06737 (10)	1.43143 (14)	0.0490 (5)	
C3	−0.27405 (13)	−0.01485 (10)	1.50880 (12)	0.0425 (4)	
C4	−0.25865 (15)	0.08194 (11)	1.50733 (15)	0.0514 (5)	
C5	−0.16973 (14)	0.12463 (10)	1.42932 (15)	0.0486 (4)	
C6	−0.09233 (11)	0.07103 (8)	1.34970 (11)	0.0351 (3)	
C7	−0.37282 (16)	−0.06253 (12)	1.59045 (16)	0.0589 (5)	
C8	−0.00193 (12)	0.11530 (8)	1.26170 (11)	0.0346 (3)	
C11	0.10936 (12)	0.14692 (8)	1.07341 (12)	0.0352 (3)	
C12	0.07217 (12)	0.06513 (8)	1.15977 (11)	0.0358 (3)	
C14	0.24379 (12)	0.14657 (9)	1.02232 (12)	0.0374 (3)	
C16	0.45719 (14)	0.13194 (11)	1.04688 (14)	0.0498 (5)	
C17	0.42854 (13)	0.15256 (9)	0.91647 (13)	0.0434 (4)	
C18	0.28814 (13)	0.16133 (10)	0.90353 (13)	0.0431 (4)	
C19	0.58217 (16)	0.11677 (16)	1.09931 (19)	0.0757 (7)	
C20	0.68147 (16)	0.12359 (15)	1.0141 (2)	0.0747 (7)	
C21	0.65735 (16)	0.14460 (13)	0.8845 (2)	0.0670 (6)	
C22	0.53186 (17)	0.15950 (12)	0.83303 (17)	0.0608 (6)	
H1	−0.05400	−0.06330	1.30160	0.0530*	
H2	−0.20290	−0.13250	1.43210	0.0590*	
H4	−0.30920	0.11890	1.56000	0.0620*	
H5	−0.16150	0.18970	1.43000	0.0580*	
H7A	−0.44440	−0.08530	1.53470	0.0880*	
H7B	−0.40510	−0.01840	1.65190	0.0880*	
H7C	−0.33190	−0.11430	1.63680	0.0880*	
H12A	0.01720	0.02000	1.11180	0.0430*	
H12B	0.14910	0.03350	1.19800	0.0430*	
H13	0.02420	0.20390	0.93240	0.0640*	
H18	0.23800	0.17470	0.82740	0.0520*	
H19	0.59860	0.10270	1.18730	0.0910*	
H20	0.76740	0.11370	1.04540	0.0900*	
H21	0.72720	0.14890	0.83020	0.0800*	
H22	0.51630	0.17380	0.74500	0.0730*	

### Atomic displacement parameters (Å^2^)

**Table:**

	*U*^11^	*U*^22^	*U*^33^	*U*^12^	*U*^13^	*U*^23^
O10	0.0539 (6)	0.0394 (5)	0.0492 (5)	−0.0152 (4)	0.0203 (4)	−0.0082 (4)
O13	0.0359 (5)	0.0471 (5)	0.0441 (5)	−0.0045 (4)	0.0002 (4)	0.0092 (4)
O15	0.0352 (5)	0.0961 (8)	0.0367 (5)	−0.0054 (5)	0.0013 (4)	0.0150 (5)
N9	0.0475 (6)	0.0416 (6)	0.0451 (6)	−0.0103 (5)	0.0175 (5)	−0.0081 (4)
C1	0.0504 (8)	0.0395 (7)	0.0445 (7)	0.0007 (5)	0.0132 (6)	−0.0021 (5)
C2	0.0581 (9)	0.0389 (7)	0.0508 (8)	−0.0041 (6)	0.0115 (7)	0.0050 (6)
C3	0.0395 (7)	0.0531 (8)	0.0351 (6)	−0.0041 (5)	0.0029 (5)	0.0083 (5)
C4	0.0497 (8)	0.0542 (8)	0.0522 (8)	−0.0015 (6)	0.0213 (7)	−0.0052 (6)
C5	0.0519 (8)	0.0382 (6)	0.0576 (8)	−0.0040 (6)	0.0204 (7)	−0.0060 (6)
C6	0.0332 (6)	0.0400 (6)	0.0322 (6)	−0.0031 (5)	0.0033 (5)	−0.0007 (5)
C7	0.0548 (9)	0.0719 (10)	0.0509 (8)	−0.0084 (7)	0.0120 (7)	0.0170 (7)
C8	0.0340 (6)	0.0375 (6)	0.0323 (6)	−0.0035 (5)	0.0029 (5)	−0.0026 (4)
C11	0.0346 (6)	0.0371 (6)	0.0340 (6)	−0.0041 (5)	0.0035 (5)	−0.0021 (4)
C12	0.0385 (6)	0.0357 (6)	0.0337 (6)	−0.0023 (5)	0.0064 (5)	−0.0014 (5)
C14	0.0348 (6)	0.0435 (6)	0.0338 (6)	−0.0034 (5)	0.0012 (5)	0.0015 (5)
C16	0.0354 (7)	0.0679 (9)	0.0464 (8)	−0.0041 (6)	0.0055 (6)	0.0068 (6)
C17	0.0405 (7)	0.0481 (7)	0.0422 (7)	0.0003 (5)	0.0085 (6)	−0.0001 (5)
C18	0.0394 (7)	0.0565 (8)	0.0337 (6)	0.0036 (6)	0.0040 (5)	0.0018 (5)
C19	0.0392 (9)	0.1234 (17)	0.0637 (11)	−0.0029 (9)	−0.0044 (8)	0.0191 (11)
C20	0.0354 (8)	0.0994 (14)	0.0893 (14)	−0.0008 (8)	0.0036 (8)	0.0033 (11)
C21	0.0447 (9)	0.0773 (12)	0.0813 (12)	−0.0022 (7)	0.0260 (8)	−0.0039 (9)
C22	0.0543 (9)	0.0773 (11)	0.0528 (9)	0.0024 (8)	0.0203 (7)	0.0011 (8)

### Geometric parameters (Å, º)

**Table:**

O10—N9	1.4254 (15)	C16—C17	1.384 (2)
O10—C11	1.4597 (15)	C17—C18	1.4394 (19)
O13—C11	1.3917 (15)	C17—C22	1.397 (2)
O15—C14	1.3739 (16)	C19—C20	1.377 (3)
O15—C16	1.3739 (17)	C20—C21	1.371 (3)
O13—H13	0.8200	C21—C22	1.377 (2)
N9—C8	1.2808 (16)	C1—H1	0.9300
C1—C2	1.385 (2)	C2—H2	0.9300
C1—C6	1.3869 (17)	C4—H4	0.9300
C2—C3	1.379 (2)	C5—H5	0.9300
C3—C4	1.386 (2)	C7—H7A	0.9600
C3—C7	1.507 (2)	C7—H7B	0.9600
C4—C5	1.383 (2)	C7—H7C	0.9600
C5—C6	1.3935 (19)	C12—H12A	0.9700
C6—C8	1.4671 (16)	C12—H12B	0.9700
C8—C12	1.5028 (17)	C18—H18	0.9300
C11—C14	1.4961 (18)	C19—H19	0.9300
C11—C12	1.5225 (17)	C20—H20	0.9300
C14—C18	1.3380 (18)	C21—H21	0.9300
C16—C19	1.376 (2)	C22—H22	0.9300
			
N9—O10—C11	108.67 (9)	C16—C19—C20	116.20 (17)
C14—O15—C16	105.83 (10)	C19—C20—C21	121.95 (16)
C11—O13—H13	109.00	C20—C21—C22	121.36 (16)
O10—N9—C8	108.96 (10)	C17—C22—C21	118.27 (16)
C2—C1—C6	120.35 (12)	C2—C1—H1	120.00
C1—C2—C3	121.91 (13)	C6—C1—H1	120.00
C2—C3—C4	117.54 (13)	C1—C2—H2	119.00
C4—C3—C7	122.23 (13)	C3—C2—H2	119.00
C2—C3—C7	120.24 (13)	C3—C4—H4	119.00
C3—C4—C5	121.42 (14)	C5—C4—H4	119.00
C4—C5—C6	120.61 (13)	C4—C5—H5	120.00
C1—C6—C5	118.16 (11)	C6—C5—H5	120.00
C1—C6—C8	120.44 (11)	C3—C7—H7A	109.00
C5—C6—C8	121.38 (11)	C3—C7—H7B	109.00
N9—C8—C6	121.81 (11)	C3—C7—H7C	109.00
N9—C8—C12	112.70 (11)	H7A—C7—H7B	109.00
C6—C8—C12	125.48 (10)	H7A—C7—H7C	109.00
O10—C11—C12	102.46 (9)	H7B—C7—H7C	109.00
O10—C11—C14	106.56 (10)	C8—C12—H12A	112.00
O10—C11—O13	110.77 (9)	C8—C12—H12B	112.00
O13—C11—C14	111.19 (10)	C11—C12—H12A	112.00
C12—C11—C14	117.60 (10)	C11—C12—H12B	112.00
O13—C11—C12	107.88 (10)	H12A—C12—H12B	109.00
C8—C12—C11	101.05 (9)	C14—C18—H18	127.00
O15—C14—C18	111.78 (11)	C17—C18—H18	127.00
C11—C14—C18	132.83 (12)	C16—C19—H19	122.00
O15—C14—C11	115.38 (10)	C20—C19—H19	122.00
O15—C16—C17	110.29 (12)	C19—C20—H20	119.00
C17—C16—C19	123.67 (14)	C21—C20—H20	119.00
O15—C16—C19	126.04 (14)	C20—C21—H21	119.00
C16—C17—C18	105.42 (12)	C22—C21—H21	119.00
C18—C17—C22	136.03 (13)	C17—C22—H22	121.00
C16—C17—C22	118.55 (13)	C21—C22—H22	121.00
C14—C18—C17	106.68 (12)		
			
C11—O10—N9—C8	13.54 (13)	C6—C8—C12—C11	161.79 (11)
N9—O10—C11—O13	91.49 (11)	O10—C11—C12—C8	23.18 (11)
N9—O10—C11—C12	−23.35 (12)	O13—C11—C12—C8	−93.75 (11)
N9—O10—C11—C14	−147.45 (9)	C14—C11—C12—C8	139.58 (11)
C16—O15—C14—C11	−179.68 (12)	O10—C11—C14—O15	66.25 (13)
C16—O15—C14—C18	−0.19 (16)	O10—C11—C14—C18	−113.10 (16)
C14—O15—C16—C17	0.43 (16)	O13—C11—C14—O15	−172.95 (11)
C14—O15—C16—C19	−179.12 (17)	O13—C11—C14—C18	7.7 (2)
O10—N9—C8—C6	−175.99 (10)	C12—C11—C14—O15	−47.91 (16)
O10—N9—C8—C12	3.06 (14)	C12—C11—C14—C18	132.74 (15)
C6—C1—C2—C3	0.0 (2)	O15—C14—C18—C17	−0.11 (16)
C2—C1—C6—C5	1.11 (19)	C11—C14—C18—C17	179.27 (13)
C2—C1—C6—C8	−177.02 (12)	O15—C16—C17—C18	−0.49 (16)
C1—C2—C3—C4	−1.2 (2)	O15—C16—C17—C22	179.71 (13)
C1—C2—C3—C7	178.48 (13)	C19—C16—C17—C18	179.07 (17)
C2—C3—C4—C5	1.3 (2)	C19—C16—C17—C22	−0.7 (2)
C7—C3—C4—C5	−178.39 (14)	O15—C16—C19—C20	179.82 (17)
C3—C4—C5—C6	−0.2 (2)	C17—C16—C19—C20	0.3 (3)
C4—C5—C6—C1	−1.0 (2)	C16—C17—C18—C14	0.36 (16)
C4—C5—C6—C8	177.08 (13)	C22—C17—C18—C14	−179.89 (16)
C1—C6—C8—N9	−174.48 (12)	C16—C17—C22—C21	0.6 (2)
C1—C6—C8—C12	6.60 (18)	C18—C17—C22—C21	−179.14 (16)
C5—C6—C8—N9	7.46 (19)	C16—C19—C20—C21	0.2 (3)
C5—C6—C8—C12	−171.46 (12)	C19—C20—C21—C22	−0.3 (3)
N9—C8—C12—C11	−17.21 (13)	C20—C21—C22—C17	−0.1 (3)

### Hydrogen-bond geometry (Å, º)

**Table:**

*D*—H···*A*	*D*—H	H···*A*	*D*···*A*	*D*—H···*A*
O13—H13···N9^i^	0.82	2.17	2.9352 (15)	156
C2—H2···O10^ii^	0.93	2.46	3.2328 (17)	141
C7—H7*C*···O15^iii^	0.96	2.58	3.175 (2)	121
C18—H18···O10^i^	0.93	2.54	3.4183 (17)	158

Symmetry codes: (i) *x*, −*y*+1/2, *z*−1/2; (ii) −*x*, *y*−1/2, −*z*+5/2; (iii) −*x*, −*y*, −*z*+3.
