# Supplementary figures and images for: Crystal structure of 5-(1-benzo­furan-2-yl)-3-(4-methyl­phen­yl)-4,5-di­hydro-1,2-oxazol-5-ol
Source: Acta Crystallogr E Crystallogr Commun. 2015 Jun 17;71(Pt 7):o492–3. doi: 10.1107/S2056989015011263 (PMC4518973; doi:10.1107/S2056989015011263)

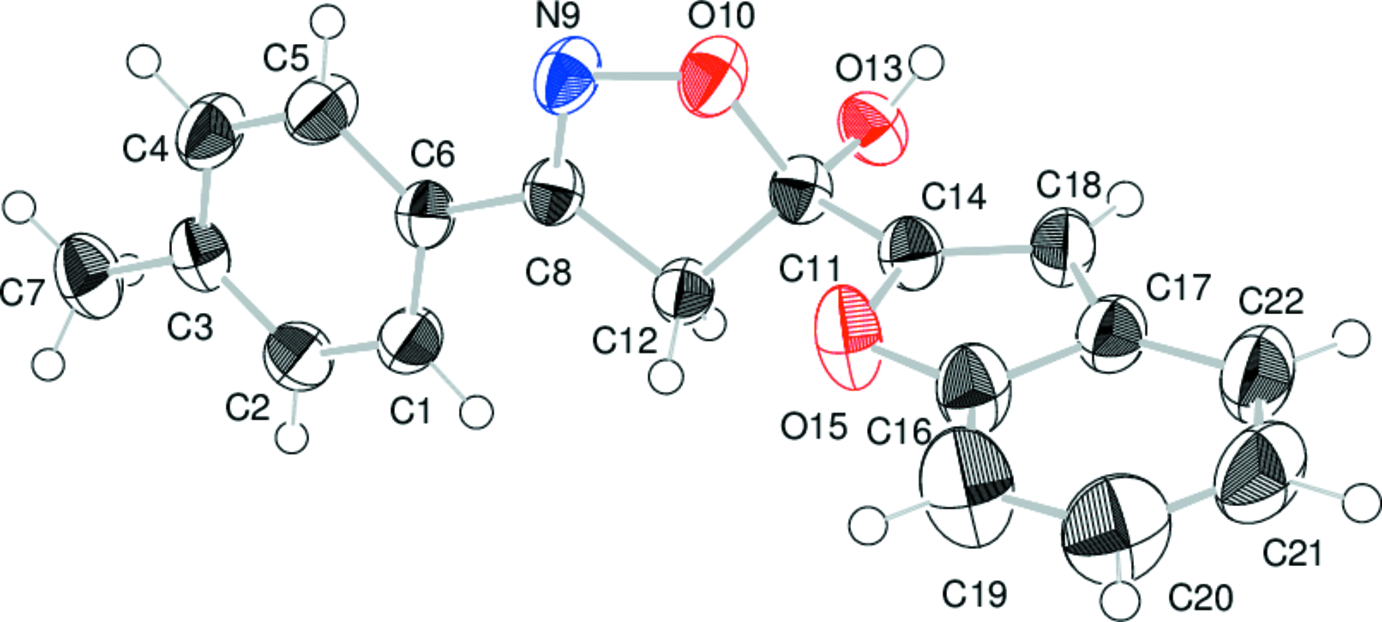

Supplement: Supplementary file 4 [file e-71-0o492-fig1.tif]

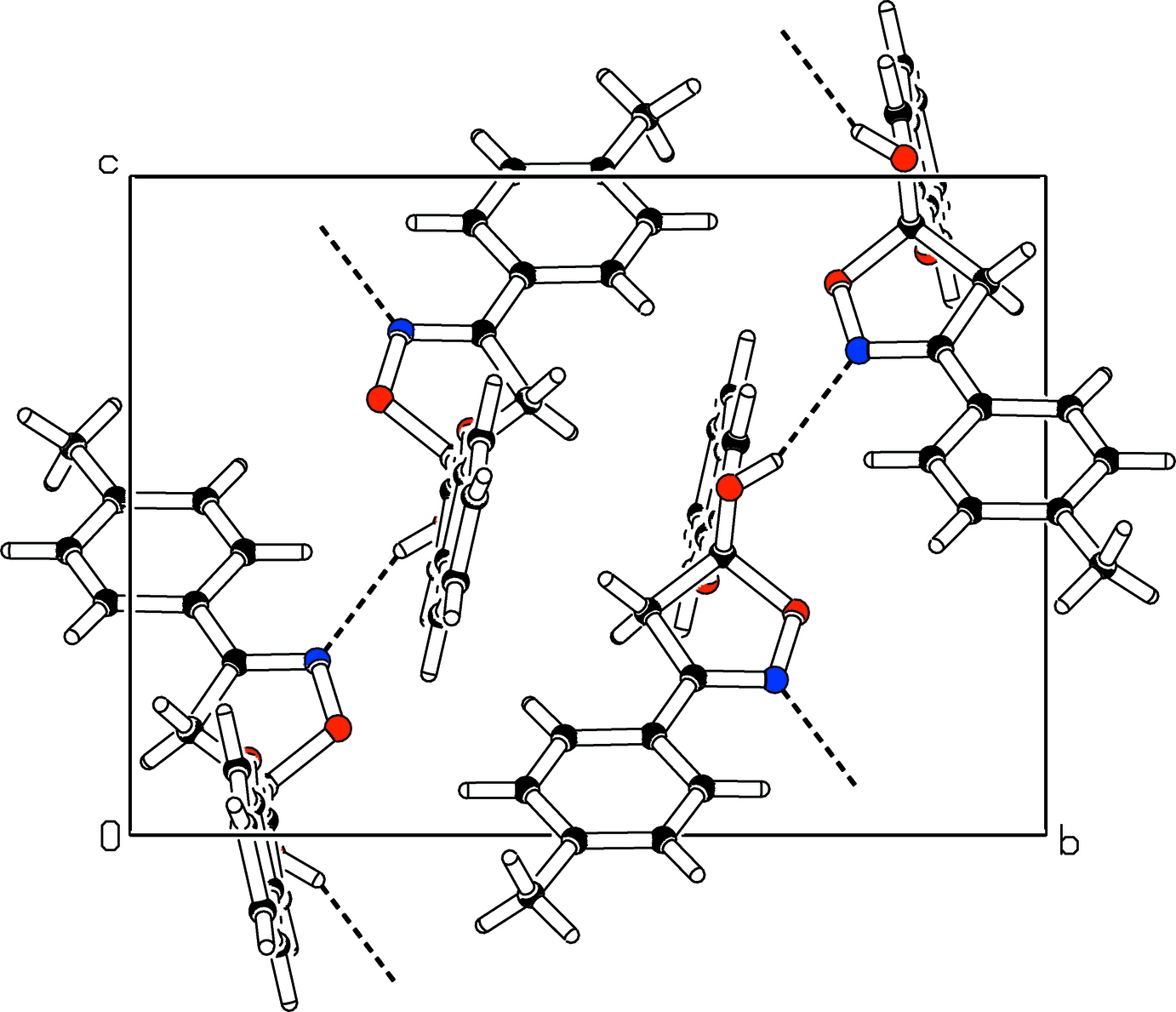

Supplement: Supplementary file 5 [file e-71-0o492-fig2.tif]
